# Biologics Reduce Symptoms of Alcohol Intolerance Better than Aspirin Desensitization in Patients with N-ERD and Nasal Polyps

**DOI:** 10.3390/biomedicines12051025

**Published:** 2024-05-07

**Authors:** Ulrike Foerster-Ruhrmann, Miroslav Jurkov, Agnieszka J. Szczepek, Karl-Christian Bergmann, Joachim W. Fluhr, Heidi Olze

**Affiliations:** 1 Department of Otorhinolaryngology, Head and Neck Surgery, Charité-Universitätsmedizin Berlin, Corporate Member of Freie Universität and Berlin Humboldt Universität zu Berlin, Charitéplatz 1, 10117 Berlin, Germany; ulrike.foerster@charite.de (U.F.-R.); dr.jurkov@yahoo.com (M.J.); agnes.szczepek@charite.de (A.J.S.); 2 Institute of Allergology, Charité—Universitätsmedizin Berlin, Corporate Member of Freie Universität Berlin and Humboldt-Universität zu Berlin, 12203 Berlin, Germany; karl-christian.bergmann@charite.de (K.-C.B.); joachim.fluhr@charite.de (J.W.F.); 3 Fraunhofer Institute for Translational Medicine and Pharmacology, Immunology and Allergology, 12203 Berlin, Germany

**Keywords:** non-steroidal anti-inflammatory drugs (NSAIDs) exacerbated respiratory disease (N-ERD), chronic rhinosinusitis (CRS), chronic rhinosinusitis with nasal polyps (CRSwNP), biologics, acetylsalicylic acid (ASA) desensitization, alcohol intolerance

## Abstract

Background: Non-steroidal anti-inflammatory drugs (NSAIDs) exacerbated respiratory disease (N-ERD) is associated with chronic rhinosinusitis with nasal polyps (CRSwNP), asthma, and NSAID hypersensitivity. An overproduction of leukotrienes characterizes the pathomechanism of the disease. N-ERD patients often report breathing difficulties after consuming alcohol. These symptoms have been observed in patients receiving either aspirin therapy after desensitization (ATAD), therapy with the biologics dupilumab (anti-IL-4Ra antibody) and omalizumab (anti-IgE antibody), or intranasal corticosteroid treatment (INCS). Methods: This retrospective, real-world study assessed the severity of alcohol-related and non-alcohol-related respiratory symptoms in CRSwNP/N-ERD patients 3–6 months after ATAD, biologic (dupilumab or omalizumab), or INCS therapy. A total of 171 patients (98 women and 73 men) were enrolled in the study. All groups received standard INCS therapy. Sixty-three patients were treated with ATAD; 48 received biologics (dupilumab *n* = 31; omalizumab *n* = 17); and 60 received INCS only and served as a control group. Alcohol-dependent symptoms and typical CRS symptoms (alcohol-independent) were quantified using visual analog scales (VAS). Results: ATAD and biological therapy significantly reduced VAS scores for alcohol dependence and CRS symptoms. In the control group receiving INCS, only non-alcohol dependent CRS symptoms improved significantly (*p* < 0.05). The most significant differences in pre/post scores were observed in patients receiving dupilumab, with the most significant improvement in alcohol-dependent and CRS symptoms (dupilumab > omalizumab > ATAD). Conclusions: This real-world study shows that alcohol-related respiratory symptoms are a relevant parameter in CRSwNP/N-ERD patients. Patients benefit more from biologic therapy than from ATAD in terms of their alcohol-related symptoms and other CRS symptoms. Future studies should include placebo-controlled oral alcohol challenge.

## 1. Introduction

Consumption of alcohol is not free of risks [1]. Moderate drinking (2–4 drinks during the day) was shown to be associated with an immediate higher cardiovascular risk, whereas heavy drinking (6–9 drinks a day or 19–30 drinks a week) significantly increased the risk for myocardial infarction and hemorrhagic stroke on the following day [2]. Alcohol consumption has been shown to increase the rate of breast cancer in women [3], and other types of neoplasia in the general population, such as liver, esophageal, and stomach cancers [4]. Notably, alcohol consumption has been associated with adverse cognitive and personality alterations in adolescents [5] and adults [6]. Occasionally, even low-dose alcohol consumption can cause death [7].

One of the rare adverse reactions to alcohol is alcohol-related respiratory symptoms. In the general European population, the prevalence of alcohol-related symptoms ranges from 3.4 to 7.6%, based on questionnaire-based studies. Red wine was the most common trigger of adverse reactions [8,9,10]. Alcohol-related responses are generally not specific to one type of alcoholic beverage and often occur after drinking small amounts of alcohol [11,12]. Nasal congestion, sneezing, runny nose, flushing, and lower respiratory symptoms have been reported after drinking alcoholic beverages [8,9,10]. Fatal reactions after alcohol consumption have also been reported [7]. The specific components of alcoholic beverages causing respiratory symptoms remain unknown because they are complex, consisting of hundreds of components in addition to ethanol. The phenolic compounds, histamine, sulfites, or yeast, have been implicated in sensitivity to wine or beer [13,14,15]. These components play an essential role in determining the flavor and character of alcoholic beverages [13]. Prick tests to their constituents (ethanol, acetic acid, or acetaldehyde) or to wine and beer were negative in patients with a history of alcoholic reactions, suggesting non-allergic sensitivity [16]. Epidemiological studies of alcohol-related respiratory responses in patients with asthma have shown a prevalence of approximately 33% [17,18]. The association between alcohol-related asthma and asthma intolerant to non-steroidal anti-inflammatory drugs (NSAIDs) has also been demonstrated, suggesting a common underlying mechanism for these adverse reactions [13,17].

NSAIDs-exacerbated respiratory disease (N-ERD) [15] is a distinct clinical entity characterized by asthma, chronic rhinosinusitis with nasal polyps (CRSwNP), and respiratory reactions following NSAID intake. N-ERD prevalence in the general population is 0.6–2.5%; however, it occurs in 4.3–21% of asthma patients and 15–40% in subjects with asthma and CRSwNP [19,20]. N-ERD is characterized by severe type 2 airway inflammation, extensive eosinophilic infiltration, and ongoing mast cell activation [19,21,22] There is an imbalance between the overproduction of proinflammatory and bronchoconstrictor eicosanoids, especially leukotriene (LT) C_4_, as a consequence of an impaired cyclooxygenase (COX) enzyme pathway [23,24] and levels of COX-2 and prostaglandin (PG) E_2_ are diminished [19,25,26]. A non-allergic mechanism underlying the precipitation of asthmatic attacks by COX-1 inhibitors such as acetylsalicylic acid (ASA) in hypersensitive patients was proposed [24]. Reactions in respiratory airways were associated with mast cell activation, histamine release, tryptase, and LTs [25]. 

Chronic rhinosinusitis (CRS) is defined as the presence of the symptoms of nasal blockage or anterior/posterior nasal drip, facial pain, and loss of smell for 12 or more weeks. There are two phenotypes of CRS: CRS without nasal polyps CRSsNP and CRS with nasal polyps CRSwNP [27]. The prevalence of N-ERD has been reported to be 25–32% in patients with CRSwNP [28]. Recurrent nasal polyps or severe asthma often impair the quality of life in N-ERD patients. They also report quality-of-life-impairing respiratory symptoms such as runny nose, itching, sneezing, or asthma attacks immediately after drinking alcohol [11,12,16,29,30,31]. 

Therapeutic options for CRSwNP with N-ERD include nasal sinus surgeries, corticosteroids, aspirin therapy after desensitization (ATAD) [32], and currently, biologics [27,28,33,34,35,36,37,38]. So far, small clinical studies have shown that ATAD [39], dupilumab (anti-IL-4Ra antibody inhibiting signaling of IL-4 and IL-13) [28,40], omalizumab (anti-IgE antibody) [41,42], and histamine and leukotriene receptor antagonists improve respiratory alcohol-induced symptoms [43]. Still, the severity of alcohol-related respiratory symptoms in CRSwNP patients has not been systematically investigated and compared so far. 

This retrospective study, which was based on real-life data, was prompted by our clinical observations and reports from CRSwNP patients. The study aimed to determine if the CRSwNP-oriented therapy influences the alcohol-intolerance-related symptoms found in this group. 

The primary outcome measure was the assessment of the severity of respiratory alcohol-induced symptoms in CRSwNP patients with N-ERD before and after 3–6 months of therapy with ATAD, dupilumab, omalizumab, or intranasal corticosteroids only (INCS, controls). The secondary outcome measure was the assessment of the severity of typical CRS symptoms (alcohol-independent) before and after 3–6 months of therapy.

## 2. Materials and Methods

### 2.1. Patients

Patients with a positive history of respiratory alcohol reactions and an endoscopically confirmed CRSwNP phenotype were included between 2013 and 2023. Patients were recruited consecutively from the ORL Department and the Allergy Center. All patients were warned about the possible adverse effects of alcohol on their general health during the interview. Despite these warnings, all patients enrolled in the study chose to drink occasionally (e.g., to celebrate birthdays, weddings, or New Year’s parties).

### 2.2. Inclusion Criteria

Patients were included if they were ≥18 years of age, had a confirmed diagnosis of CRSwNP and N-ERD disease according to the EPOS criteria [27,44], and had a positive history of respiratory symptoms such as nasal obstruction, runny nose, postnasal drip, sneezing, or general asthmatic symptoms immediately after alcohol consumption. Patients were enrolled if they received first-line standard therapy with ATAD, dupilumab, omalizumab, or intranasal corticosteroid therapy (INCS) alone (controls). All patients received INCS as the standard of care. 

### 2.3. Exclusion Criteria

Patients were excluded if they had no history of positive airway response to alcohol, did not voluntarily report their alcohol consumption, or had discontinued ATAD or biologics within the previous 3–6 months. Multiple therapies of ATAD or biologics were excluded.

### 2.4. Ethics

The local ethics committee approved this retrospective monocentric study (permit number EA1-245-22 obtained on 6 April 2023).

### 2.5. Study Design

#### 2.5.1. Study Population

The severity of respiratory symptoms before and after 3–6 months of standard therapy with ATAD, biologics, or intranasal corticosteroids alone was evaluated retrospectively in all patients. Age, sex, specific type of intolerance (e.g., to which type of alcohol do patients react), presence of CRSwNP, number of nasal sinus surgeries for nasal polyps, NSAID-exacerbated respiratory disease (N-ERD) according to positive history of NSAID intolerance and/or nasal/oral acetylsalicylic acid (ASA) challenge [44], and presence of asthma are summarized in Table 1. Asthma diagnoses were made by outpatient pulmonologists [45].

This retrospective study included 171 patients who received either daily oral acetylsalicylic acid (ASA) after ATAD, the biologics dupilumab or omalizumab, or INCS alone (controls) (Table 1). Patients desensitized to ASA received 300 mg of ASA as a daily maintenance dose. Dupilumab and omalizumab were administered according to standard manufacturer protocols.

#### 2.5.2. Diagnosis of N-ERD and ASA Desensitization Protocol

In the 63 patients treated with ATAD, the diagnosis of N-ERD was based on an oral aspirin challenge [46]. Challenges were combined with a desensitization procedure, for which the patients were admitted for three days to the ENT hospital ward. The procedure was based on a modified published protocol [47], following the recommendations of the European Academy of Allergy and Clinical Immunology (EAACI) [44]. On the first day, each patient received an oral placebo at 8 a.m., followed by 5 mg of acetylsalicylic acid (ASA) at 11 a.m., 25 mg of ASA at 2 p.m., and 50 mg of ASA at 5 p.m. The patients were continuously monitored, and the challenge was discontinued in case of an asthma attack, but not if the challenge induced only nasal symptoms. On the second day, the patients were challenged with 50 mg ASA at 7 a.m., 75 mg ASA at 11 a.m., 150 mg ASA at 1 p.m., and 300 mg ASA at 4 p.m. On the third day, the patients were challenged only with one dose of ASA (300 mg) at 8 a.m. and discharged at 1 p.m. All patients were instructed to take 300 mg of ASA daily to maintain the tolerance achieved. In the remaining patients, the diagnosis of N-ERD was based on medical history, strictly following the EAACI guidelines [44].

#### 2.5.3. Evaluation of Alcohol-Dependent Symptoms with Visual Analog Scales

Patients were asked which type of alcohol (beer, white wine, red wine, sparkling wine, liqueur, or other alcohol) they observed respiratory nasal and bronchial symptoms. Multiple responses were allowed (Table 1).

Changes in respiratory symptoms occurring immediately after the consumption of alcohol before and after 3–6 months of therapy were scored retrospectively by the patients using visual analog scales (VAS) for any or a combination of nasal symptoms such as nasal congestion, runny nose, postnasal drip, sneezing, and asthma symptoms, with a score from 0 to 100 (maximum symptoms) [27].

#### 2.5.4. Evaluation of CRS Symptoms (Alcohol-Independent) with Visual Analog Scales 

Changes in the typical persistent CRS symptoms of nasal congestion, runny nose, postnasal drip, loss of smell, and facial pain according to the CRS definition of EPOS criteria (European Position Paper on Rhinosinusitis and Nasal Polyps) criteria [27] before and after 3–6 months of treatment were retrospectively scored by the patients using visual analog scales (VAS) with a maximum score of 100. 

### 2.6. Statistics

The Kolmogorov–Smirnov test showed that the data for most variables were not normally distributed. Therefore, nonparametric analyses were applied. The exact Wilcoxon test was used to compare VAS scores for alcohol-dependent and alcohol-independent symptoms before and after 3–6 months of treatment (*p* < 0.05). An exploratory factor analysis (method: principal component analysis) was calculated. Based on the bivariate Pearson correlation matrix, four characteristics were distinguished separately for each block: (1) Alcohol-dependent symptoms before therapy; (2) alcohol-dependent symptoms after therapy; (3) CRS symptoms independent of alcohol before therapy, (4) CRS symptoms independent of alcohol after therapy. Kaiser–Meyer–Olkin (KMO) and Bartlett’s sphericity tests were performed. Only one factor with an eigenvalue > 1 was extracted in each case. For each patient, the mean of the values from the measured initial characteristics of the individual blocks was calculated as the new block factor. The scales from 0 to 100 are thus retained. 

## 3. Results

### 3.1. Evaluation of Alcohol-Dependent Reactions 

**Figure 1:**
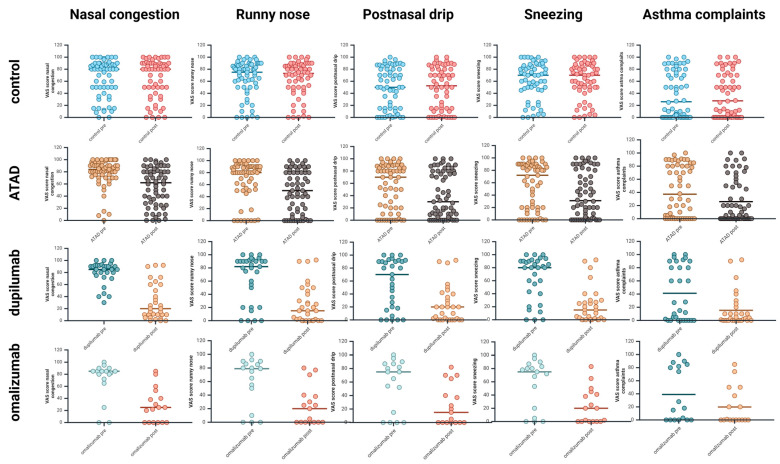
Alcohol-induced respiratory symptoms in patients with CRSwNP before and after treatment. All symptoms were measured by VAS and remained unchanged after therapy in the control group (INCS), but improved significantly in the ATAD group and the groups treated with dupilumab or omalizumab. Individual data points are shown for each therapy group before and after therapy, with the median marked by a vertical line. CRSwNP, chronic rhinosinusitis with nasal polyps; ATAD, aspirin therapy after desensitization; INCS, intranasal corticosteroids. Created with Biorender.com.

After 3–6 months of therapy, the alcohol-induced respiratory symptoms such as nasal congestion, runny nose, postnasal drip, sneezing, and asthma complaints were significantly reduced in the groups ATAD, dupilumab, and omalizumab (*p* ≤ 0.001, exact two-sided Wilcoxon test for matched pairs). In contrast, no significant changes were found for the INCS control group (Figure 1). 

The typical, alcohol-independent CRS symptoms decreased significantly in all groups, including the control INCS group (*p* ≤ 0.001 for all groups (Table 2). 

### 3.2. Exploratory Factor Analysis of Alcohol-Induced Respiratory Symptoms and CRS Symptoms (Alcohol-Independent)

**Figure 2:**
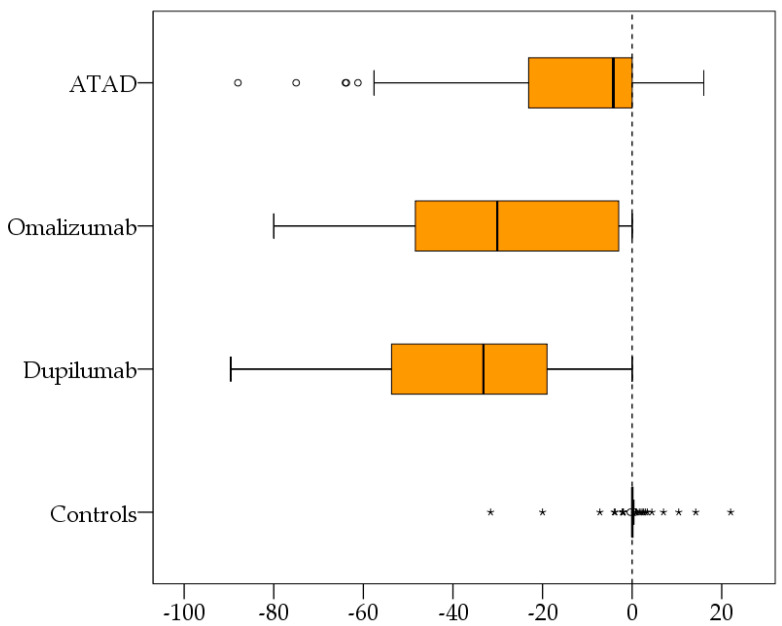
Box plots (generated with SPSS) for the pairwise differences after 3–6 months of therapy and before therapy of the recalculated factor “alcohol-related complaints” show a significant symptom reduction with each treatment, with the most striking differences ΔF for dupilumab > omalizumab > ATAD. No change was observed in the control group. ATAD, aspirin therapy after desensitization. * extreme values; ° outlier values.

Each block’s factor analysis was calculated separately using the Pearson correlation matrix. The mean value of all bivariate Pearson correlations within blocks was 0.574. The average value of the Kaiser–Meyer–Olkin (KMO) test was 0.85. The average proportion of variance explained was 65.4%. From the characteristics of each block, the mean of the values was calculated as a new block factor. This reduced the number of characteristics to be analyzed to these four block factors (as described above):

Pairwise factor differences ΔF: Fx after 3–6 months of therapy minus Fx before treatment was used to measure the effect of the different therapies (see Table 1). The general factor of exploratory factor analysis, “complaints after alcohol” with differences before and after 3–6 months, achieved a significant reduction for each treatment group and showed the most prominent differences ΔF for dupilumab > omalizumab > ASA (ATAD) without significant changes for the control group with intranasal corticosteroids (Figure 2).

**Figure 3:**
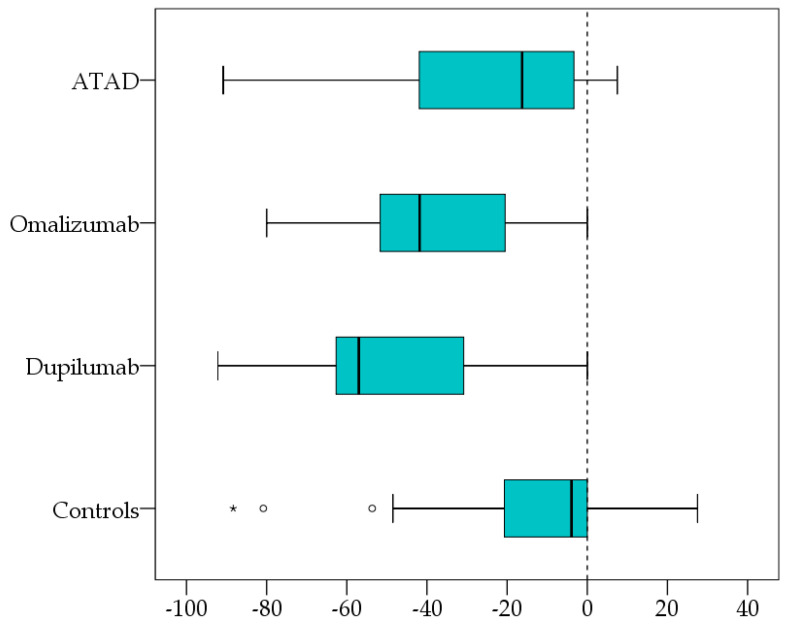
Box plots (generated with SPSS) for the pairwise differences before and after 3–6 months of therapy of the recalculated factor “CRS complaints” (alcohol-independent) show a significant symptom reduction for each treatment with the most striking differences ΔF for dupilumab > omalizumab > ATAD. ATAD, aspirin therapy after desensitization. CRS: chronic rhinosinusitis. Improvement may also be observed in controls (nasal cortisone spray only). * extreme values; ° outlier values.

The general factor of exploratory factor analysis, “CRS complaints” (alcohol-independent), with differences before and after 3–6 months of therapy, showed the most significant differences ΔF for dupilumab > omalizumab > ASA. (ATAD) (Figure 3). Each treatment group achieved significant reductions in alcohol-independent CRS symptoms. A significant improvement was also observed in the control group (nasal cortisone spray only). However, this is less pronounced than with the other treatments.

## 4. Discussion

This retrospective study of real-world data is the first to examine the severity of alcohol-induced respiratory symptoms before and after 3–6 months of treatment with ATAD, dupilumab, omalizumab, or INCS. It showed that the three first treatments, but not INCS, effectively reduced alcohol-induced respiratory symptoms in patients with CRSwNP and N-ERD (Figure 1 and Figure 2). The most remarkable improvement was demonstrated in the patients treated with dupilumab, followed by omalizumab and ATAD (Figure 2). 

Alcohol affects the innate and adaptive immune system in several ways, including skewing the immune response towards the eosinophilic T2 endotype [48]. Alcohol-induced respiratory symptoms are common in patients with N-ERD; however, the pathomechanism of alcohol-dependent symptoms has not yet been clarified. 

Cardet et al. studied patients based on medical history in the following patient groups: N-ERD (*n* = 59), ASA-tolerant group (*n* = 54), CRS (*n* = 50), and healthy controls (*n* = 50) and found that the proportion of respiratory alcohol-induced symptoms was highest in the N-ERD group with 75%, followed by 33% in the ASA-tolerant group, 30% in the CRS group, and 14% in healthy controls. The proportion of nasal polyps varied: 100% in the N-ERD group, 25% in the ASA-tolerant group, 50% in the CRS group, and 0% in healthy subjects [12]. De Schryver et al. also looked at medical history. They, too, found the percentage of respiratory symptoms to alcohol highest in the N-ERD group at 63% (N-ERD group *n* = 91), followed by CRSwNP with comorbid asthma at 46% (*n* = 178), CRSwNP without asthma at 32% (*n* = 242), CRSsNP at 26% (*n* = 97), and healthy subjects at 4% (*n* = 180) [11]. In a multicenter prospective study, Glicksman et al. investigated respiratory alcohol-induced symptoms before and after at least three months of ATAD (initial daily ASA dose of 1300 mg, titrated to 325 mg ASA daily within three months). 70% of participants described ATAD therapy as helpful for alcohol-induced symptoms (29). In a case series of 7 CRSwNP patients with N-ERD, Arnold et al. also described an improvement in respiratory symptoms induced by alcohol after a minimum of 5 to a maximum of 22 months of dupilumab therapy [40]. Neither study had an assessment of the severity of respiratory symptoms induced by alcohol, as we did in our current study. After three months of omalizumab therapy, Bergmann et al. performed separate oral challenges with alcohol and ASA in a patient with severe asthma, CRSwNP, and N-ERD and demonstrated entirely negative challenge results [41].

The data from our current study suggest that biologics are more effective than ATAD in relieving respiratory symptoms in CRSwNP patients with N-ERD. This was also shown in a meta-analysis comparing different biologics and ATAD trials in an indirect head-to-head analysis. ATAD had the lowest treatment efficacy and the highest adverse effects [49]. In a retrospective study, 17 patients who had failed ATAD due to ASA side effects or lack of efficacy were switched to dupilumab therapy and showed a significant improvement in Sino-Nasal Outcome-Test-22 sinonasal parameters, endoscopic findings, and olfaction after six months of dupilumab [35].

The question of the underlying pathomechanisms of respiratory symptoms induced by alcohol remains open, as we did not investigate it in our retrospective pilot study.

Acetaldehyde-induced mast cell activation is a proposed mechanism in alcohol-induced asthma patients. Acetaldehyde dehydrogenase (ALDH) is an enzyme of the ethanol and histamine pathways [50]. Moreover, LTs were shown to mediate acetaldehyde-induced bronchial hyperresponsiveness in guinea pigs [51]. A Swedish study determined a genetic factor contributing to alcohol sensitivity (SNPs in the ALDH1b gene) and suggested that a histamine-releasing effect of acetaldehyde is a plausible biological mechanism [48].

Elevated leukotrienes have been measured in peripheral blood after oral alcohol challenge in patients with a positive history of alcohol-induced cutaneous reactions [16]. High nasal ECP levels have been measured in patients with alcohol-induced respiratory symptoms and CRSwNP [11]. A leukotriene-dependent pathomechanism has been suggested to cause hyperreactive respiratory responses in CRSwNP/N-ERD patients following alcohol consumption [11,12]. Phenols have been suggested as causative agents in N-ERD patients with alcohol-induced symptoms [14]. Polyphenols such as resveratrol from red wine inhibit the enzyme COX-1, which, like NSAIDs, can lead to acute respiratory symptoms in N-ERD patients [52]. Future studies should examine biomarkers such as leukotrienes, prostaglandins, histamine, and tryptase after a placebo-controlled oral challenge with alcohol of defined polyphenol content to investigate the pathomechanisms of alcohol-induced symptoms.

In addition, our current study showed that typical CRS symptoms (alcohol-independent) also improved significantly in CRSwNP patients after 3–6 months of ATAD or biologic therapy (Table 2). The general factor of exploratory factor analysis of CRS complaints (alcohol-independent) with differences before and after 3–6 months of treatment showed the most significant differences ΔF for dupilumab > omalizumab > ASA, Figure 2. Our results align with the meta-analysis comparing the effects of biologic therapy on CRSwNP patients in indirect head-to-head studies and in patients with severe asthma and comorbid CRSwNP [49,53,54]. 

The current pilot study did not assess side effects because it included only patients who had successfully received ATAD for at least three months or were treated with biologics.

ATAD is becoming less common with the use of biologics. Moreover, new biologics such as mepolizumab targeting IL-5, a critical cytokine underlying the etiopathology of the disease [55,56], are being introduced to treat CRwNP; however, their effects on the alcohol-induced respiratory symptoms remain unknown. This study, which is the first to systematically and retrospectively evaluate the severity of alcohol-induced respiratory symptoms after ATAD and biologic therapies, is therefore of great importance, although it has some limitations. The first pitfall is that only medical history was used, and no placebo-controlled challenges were performed. However, experienced rhinologists involved in this study collected and reviewed the data. Another pitfall was that only one time interval (3–6 months after the start of therapy) was considered. In the future, it would be interesting to know how the improvement of these therapies is shown at precise time points in the short-term course and how long the effect of either treatment lasts or whether it diminishes with long-term therapy. Finally, in this analysis, the respiratory symptoms investigated were similar for alcohol-induced and typical CRS indicators. However, a clear distinction could be made between symptoms occurring immediately after drinking and specific persistent CRS symptoms.

## 5. Conclusions

In conclusion, this study demonstrates that the response of CRSwNP/N-ERD patients to alcohol is a relevant parameter that should be addressed in future studies. To our knowledge, this is the first study assessing the severity of alcohol-related respiratory symptoms in CRSwNP/N-ERD and comparing the efficacy of different therapies. After 3–6 months of ATAD treatment or therapy with dupilumab or omalizumab, both alcohol-induced and alcohol-unrelated CRS symptoms decreased significantly. The results of this pilot study demonstrate the superiority of biologics over ATAD therapies and suggest a better outcome with dupilumab than with omalizumab. However, our results should not be used to encourage patients with CRwNP to consume alcohol but rather to improve their quality of social life occasionally. Future studies should include a placebo-controlled oral alcohol test to investigate the pathomechanism of the alcohol-induced symptoms.

## Figures and Tables

**Table 1:** Clinical characterization of the study population of patients with alcohol-dependent respiratory symptoms at the study baseline and therapy groups (ATAD, omalizumab, or dupilumab).

Sample Size	171
Age of all patients in years, mean (SD)	49.0 (12.5)
Sex female/male (*n*)	98/73
CRSwNP (*n*)	171
Asthma (*n*)	171
N-ERD (*n*)	171
Number of nasal sinus surgeries, median (min, max)	3 (range min 1; max 13)
GINA-score, median (SD)	3.42 (1.14)
Eosinophils (cells/µL), median (SD)	410 (1720.8)
Total IgE [kU/L], median (SD)	132 (342.1)
Types of therapies (*n*)	ATAD (63) Dupilumab (31) Omalizumab (17)
Control group (*n*)	Intranasal corticosteroids INCS alone (60)
Basal therapy with intranasal corticosteroids INCS (*n*)	171
Alcohol intolerance (*n*)	171
Intolerance to red wine (*n*) Intolerance to white wine (*n*) Intolerance to beer (*n*) Intolerance to sparkling wine (*n*) Intolerance to liquor (*n*) Intolerance to other alcohol (*n*)	131 111 91 91 59 54

CRSwNP, chronic rhinosinusitis with nasal polyps; N-ERD, non-steroidal anti-inflammatory drugs (NSAIDs) exacerbated respiratory disease; GINA score, Global Initiative for Asthma score; [kU/L], Kilounits per liter; ATAD, aspirin therapy after desensitization; INCS, intranasal corticosteroids; SD, standard deviation; *n*, number of patients.

**Table 2:** Typical alcohol-independent symptoms of chronic rhinosinusitis (CRS) measured by VAS pre- and post-treatment decreased significantly in each therapy group. A pairwise comparison was performed using the exact two-sided Wilcoxon test for matched pairs. ATAD, aspirin therapy after desensitization; INCS, intranasal corticosteroids; SD, standard deviation.

Median (SD)	Control INCS (*n* = 60)			ATAD (*n* = 64)			Dupilumab (*n* = 31)			Omalizumab (*n* = 17)		
	Pre	Post	*p*	Pre	Post	*p*	Pre	Post	*p*	Pre	Post	*p*
Nasal congestion	80 (27.8)	60 (33.4)	<0.001	90 (14.6)	45.5 (31.5)	<0.001	88 (14.6)	20 (24.3)	<0.001	82.5 (29.1)	25 (25.2)	<0.001
Runny nose	75 (29.6)	55 (31.4)	<0.001	80 (27.7)	42.5 (34.5)	<0.001	80 (31.4)	15 (25.2)	<0.001	82.5 (29.1)	32.5 (27)	<0.001
Postnasal drip	53 (31.8)	44.5 (32)	0.009	78.5 (32.8)	30.5 (32.9)	<0.001	80 (34)	10 (22.1)	<0.001	81 (33)	30 (27.5)	<0.001
Loss of smell	88.5 (27.5)	65 (34)	<0.001	90 (26.6)	62.5 (38.9)	<0.001	90 (23.3)	25 (33.8)	<0.001	85.5 (33.2)	22.5 (24)	<0.001
Facial pain	45 (32.5)	25 (28.3)	<0.001	60 (31)	20 (31.7)	<0.001	62 (32.5)	10 (22.5)	<0.001	80.5 (32.9)	27.5 (27.8)	<0.001

ATAD, aspirin therapy after desensitization; INCS, intranasal corticosteroids; SD, standard deviation, *p*, significance; *n*, number of patients.

## Data Availability

Data are available upon request to the corresponding author.
